# An Innovative Approach for Tailoring Molecularly Imprinted Polymers for Biosensors—Application to Cancer Antigen 15-3

**DOI:** 10.3390/bios14050222

**Published:** 2024-04-30

**Authors:** Daniela dos Santos Oliveira, Andreia Sofia Rodrigues Oliveira, Patrícia Vitorino Mendonça, Jorge Fernando Jordão Coelho, Felismina Teixeira Coelho Moreira, Maria Goreti Ferreira Sales

**Affiliations:** 1 BioMark@ISEP-CEB/LABBELS, School of Engineering, Polytechnic Institute of Porto, R. Dr. António Bernardino de Almeida, 4249-015 Porto, Portugal; ddsol@isep.ipp.pt; 2 Centre for Mechanical Engineering, Materials and Processes (CEMMPRE), Department of Chemical Engineering, Faculty of Sciences and Technology, University of Coimbra, Pole II, Rua Sílvio Lima, 3030-790 Coimbra, Portugal; uc2009108233@student.uc.pt (A.S.R.O.); patmend@eq.uc.pt (P.V.M.); 3 Instituto Pedro Nunes (IPN), Associação para a Inovação e Desenvolvimento em Ciência e Tecnologia, R. Pedro Nunes, 3030-199 Coimbra, Portugal; 4 BioMark@UC-CEB/LABBELS, Department of Chemical Engineering, Faculty of Sciences and Technology, University of Coimbra, Pole II, R. Sílvio Lima, 3030-790 Coimbra, Portugal

**Keywords:** electrochemical biosensors, atom transfer radical polymerization, cancer biomarkers, protein-imprinted polymers, gold screen-printed electrodes, cancer antigen 15-3

## Abstract

This work presents a novel approach for tailoring molecularly imprinted polymers (MIPs) with a preliminary stage of atom transfer radical polymerization (ATRP), for a more precise definition of the imprinted cavity. A well-defined copolymer of acrylamide and *N*,*N*′-methylenebisacrylamide (PAAm-co-PMBAm) was synthesized by ATRP and applied to gold electrodes with the template, followed by a crosslinking reaction. The template was removed from the polymer matrix by enzymatic/chemical action. The surface modifications were monitored via electrochemical impedance spectroscopy (EIS), having the MIP polymer as a non-conducting film designed with affinity sites for CA15-3. The resulting biosensor exhibited a linear response to CA15-3 log concentrations from 0.001 to 100 U/mL in PBS or in diluted fetal bovine serum (1000×) in PBS. Compared to the polyacrylamide (PAAm) MIP from conventional free-radical polymerization, the ATRP-based MIP extended the biosensor’s dynamic linear range 10-fold, improving low concentration detection, and enhanced the signal reproducibility across units. The biosensor demonstrated good sensitivity and selectivity. Overall, the work described confirmed that the process of radical polymerization to build an MIP material influences the detection capacity for the target substance and the reproducibility among different biosensor units. Extending this approach to other cancer biomarkers, the methodology presented could open doors to a new generation of MIP-based biosensors for point-of-care disease diagnosis.

## 1. Introduction

Cancer is the leading cause of death in the world, and its incidence continues to increase, making it the second leading cause of death in developed countries [1]. Traditional methods of cancer diagnosis include biopsy, ultrasound, and biomarker monitoring by enzyme-linked immunosorbent assay (ELISA), radioimmunoassay (RIA), electrophoresis, polymerase chain reaction (PCR), and mass spectrometry (MS) [2,3]. However, these methods have some limitations, such as long execution time and great reagent consumption, which means that there is a growing need for the early, accurate, and cost-effective detection of cancer biomarkers [4,5,6].

Biomarkers can serve as indicators for early disease diagnosis, as well as for disease prognosis. A cancer biomarker signals a normal or abnormal biological state of an organism in the form of DNA, RNA, proteins/peptides, or specific metabolites [7]. They can be detected in tissue samples and various body fluids such as urine, saliva, blood, and cerebrospinal fluid. Among several biomarkers that have been shown to be relevant or potentially relevant to cancer diseases [8,9], cancer antigen 15-3 (CA15-3) is a tumor marker for breast cancer that has clinical significance. Elevated levels of CA15-3 are detected in 10% of patients with early breast cancer, in 60% of patients with pre-surgical breast cancer, and in 80% of patients with advanced metastatic breast cancer [10].

Biosensors are now an alternative to conventional analytical methods and open up new possibilities for point-of-care (PoC) analysis [11]. Electrochemical biosensors have attracted much attention due to their simplicity, suitability, low cost, and sensitivity in PoC applications [12,13]. In this context, polyclonal/monoclonal antibodies have been used, but their corresponding biomimetic materials in the form of molecularly imprinted polymers (MIPs) have been employed as (bio)detection elements. Although the term biosensor has been reserved by IUPAC for biologically derived sensing elements, devices with MIPs were also named biosensors, maybe due to their ability to mimic antibodies [11]. They also offer advantages in terms of robustness, price, reusability, and stability [14], showing successful application to numerous cancer biomarkers [15], including biomarkers of breast cancer (such as HER-2, [16]), prostate cancer (PSA, [17]), epithelial ovarian cancer (CA-125, [18]), or hepatocellular carcinoma (AFP, [19]).

MIP materials targeting cancer biomarkers are generally established by free-radical polymerization. In this process, an initiator triggers the polymerization of monomeric and cross-linking species. The way the cross-linked polymer grows is uncontrolled and depends heavily on the concentration of the reagents, the type of the functional groups present, the temperature, and the relative amount of the chemical species, among other factors. However, the stereochemical arrangement of an imprinted site can be critical for the detection of protein biomarkers, as their intrinsic dimension and composition can change. Many cancer biomarkers are antigens that exhibit microheterogeneity and genetic polymorphism, resulting in significant differences in the overall size of glycoproteins from different sources [10]. This is the specific case of CA15-3, which means that a high degree of accuracy and control in the polymer growth/assembly phase should be considered when sizing an imprinted site for a particular cancer biomarker.

In the present work, therefore, protein imprinting was studied for the first time using well-defined copolymers with vinyl side groups that were selectively (photo)crosslinked to achieve a good match with the immobilized structure (CA 15-3). The controlled copolymers were synthesized by additional activators and reducing agents (SARAs) through atom radical transfer polymerization (ATRP) [20,21] to obtain structures that interacted with the target protein. This innovative approach offers three important advantages over conventional approaches and aims to generate structures that interact perfectly with the target protein: (a) a perfect physical/chemical interaction between the copolymers and the target protein; (b) better control over the cross-linking process to allow for the successful imprinting of the protein; (c) control over the thickness of the imprinted polymer by controlling the molecular weight of the copolymers. In the presence of the target analyte, the hydrophobic/hydrophilic/charged groups of the copolymers selectively interact with the target regions of the protein.

Overall, this work reports the development of a new generation of MIP-based materials for the production of plastic antibodies capable of selectively/efficiently detecting CA15-3, in a procedure that involves two stages of polymerization and that can be further extended to other cancer biomarkers. Considering that the preparation of suitable plastic antibodies has specific targets, well-defined copolymers with specific chain end groups were rationally designed and synthesized in a first polymerization stage. The groups present were expected to interact directly with the target biomarker, thereby contributing to the formation of a better-designed and more reproducible binding site. The copolymers were prepared by ATRP, which allowed for the fine optimization of their composition to establish important structure–property relationships. This is the first time that controlled copolymers were directly linked to the conducting segment to allow for a direct interaction with an imprinted protein cancer biomarker. A second polymerization stage was introduced to bind all polymer structures present around the target protein, which was achieved via radical polymerization with the pendant vinyl groups. The analytical features of the so-obtained MIP-based biosensors were evaluated and compared to those of a control material, prepared by conventional free-radical polymerization. The analytical features obtained were derived from calibrations in buffer and diluted serum and selectivity studies.

## 2. Material and Methods

### 2.1. Techniques

The molecular weight of the polymers was determined by size-exclusion chromatography (SEC), using an online degasser, a refractive index (RI) detector, and a set of columns (Shodex OHpak SB-G guard column, OHpak SB-804HQ, and OHpak SB-802.5HQ columns). The polymers were eluted at 0.5 mL/min with 0.1 M Na_2_SO_4_ (aq)/1 wt% acetic acid/0.02% NaN_3_ at 40 °C. Before the injection, the samples were filtered through a polytetrafluoroethylene (PTFE) membrane (0.45 μm pore size). The system was calibrated with poly(ethylene glycol) standards, and the polymer’s number-average molecular weights (*M*_n_^SEC^) and dispersity (*Ð* = *M*_w_/*M*_n_) were determined by conventional calibration using the Clarity software version 2.8.2.648.

We recorded the 400 MHz ^1^H nuclear magnetic resonance (NMR) spectra of the reaction mixture samples using a Bruker Avance III 400 MHz spectrometer (Bruker Biospin, Wissemboug, France), with a 5 mm TIX triple-resonance detection probe, in D_2_O. NMR analyses were used to confirm the chemical structure of the polymer as well as to determine the conversion of monomers, by integration of the monomer and polymer peaks using MestReNova software version: 6.0.2–5475.

The electrochemical measurements were performed in a potentiostat/galvanostat from Metrohm Autolab (Utrecht, The Netherlands), equipped with an impedimetric module and controlled by NOVA 2.1.6 software. Commercial Au-SPEs were used (Metrohm/DropSens-220AT, Oviedo, Spain), combining working and counter electrodes made of gold, and a reference electrode and electrical contacts made of silver. The switch box interfacing these SPEs were obtained from ParticleConjugation, Portugal.

Scanning electron microscopic analysis (SEM) was performed using the high-resolution (Schottky) scanning electron microscope Quanta 400 FEG ESEM/EDAX Genesis X4M (FEI Europe B.V., Eindhoven, The Netherlands).

### 2.2. Materials

All chemicals were of analytical grade, and water was deionized or of ultrapure Milli-Q laboratory grade. For the synthesis, purification, and characterization of the materials we used the following reagents: acrylamide (AAm, 99%, Acros Organics, Geel, Belgium), *N*,*N*′-methylenebisacrylamide (MBAm, 99%, Sigma-Aldrich, St. Louis, MO, USA), copper (II) chloride (CuCl_2_, 97%, Sigma-Aldrich), ethyl 2-chloropropionate (ECP, 98%, Sigma-Aldrich), *tris*[2-(dimethylamino)ethyl]amine (Me_6_TREN, 97%, Alfa Aesar, Ward Hill, MA, USA), 2,2′-Azobis[2-(2-imidazolin-2-yl)propane]dihydrochloride (VA-044, ≥98%, TCI) deuterated water (D_2_O, Eurisotop, Saint-Aubin, France), hydrochloric acid (HCl, Fisher Scientific, Waltham, MA, USA), methanol (99.8%, Eurisotop), dimethyl sulfoxide (DMSO, >99%, Sigma-Aldrich). Metallic copper (Cu^0^ wire, *d* = 1 mm, Sigma Aldrich) was washed with HCl in methanol and subsequently rinsed with methanol and dried under a stream of nitrogen, following procedures in the literature [22].

The electrochemical assays used potassium hexacyanoferrate III (K_3_[Fe(CN)_6_]) and potassium hexacyanoferrate II (K_4_[Fe(CN)_6_]) trihydrate obtained from Riedel-de Häen (Seelze, Germany); *N*-hydroxysuccinimide (NHS) and oxalic acid obtained from Merk (Darmstadt, Germany); *N*-(3-dimethyl aminopropyl)-*N*′-ethyl-carbodiimide hydrochloride (EDAC), 3-mercaptopropionic acid (3-MPA), proteinase K, and 2-aminoethyl methacrylate hydrochloride (AMA) obtained from Sigma-Aldrich; a phosphate-buffered saline (PBS, 0.01 M, pH 7.4) solution obtained from Panreac (Barcelona, Spain); fetal bovine serum (FBS) and glucose obtained from Alfa Aesar; urea obtained from Fagron (Rotterdam, The Netherlands); sulphuric acid (H_2_SO_4_) obtained from BDH (Providence, RI, USA); 2-(*N*-morpholino)ethanosulfonic acid (MES) from AppliChem (Darmstadt, Germany); carcinoembryonic antigen (CEA), 25 μg, from EastCoastBio (North Berwick, ME, USA); CA 125, 250 kU, from Hytest (Turku, Finland); and CA 15-3, 17,420 U/mL, from EmelcaBioScience (Clinge, The Netherlands).

### 2.3. Synthesis of the PAAm-co-PMBAm Copolymer and the PAAm Homopolymer

**Figure 1:**
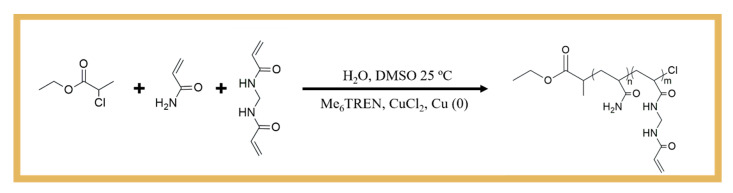

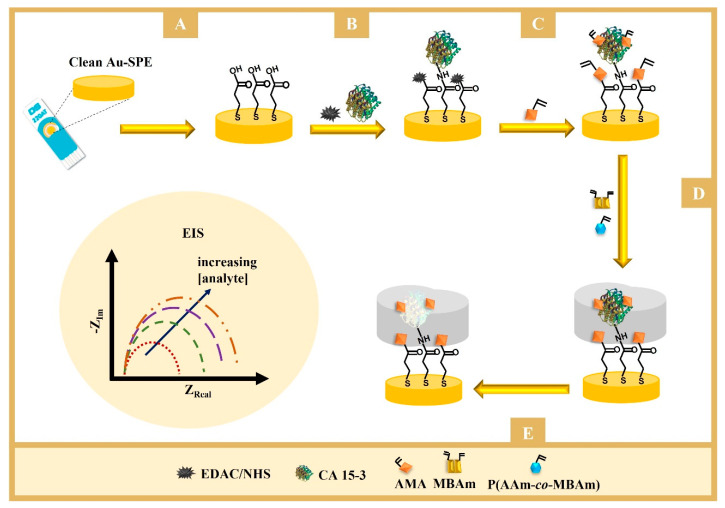
Schematic representations of (**top**) the PAAm-co-PMBAm copolymer synthesis by SARA ATRP and (**bottom**) the MIP assembly. (**A**) Au-SPE modified with thiol (3-MPA); (**B**) immobilized target molecule (CA 15-3) after activation of the carboxylic groups; (**C**) blocking of the nonspecific interaction and creation of binding sites on the protein; (**D**) polymerization with the reaction compounds around the target molecule; and (**E**) binding site formation by extraction of the target molecule with proteinase K and oxalic acid.

The synthetic approach for obtaining a well-defined poly(acrylamide)-*co*-poly(*N*,*N*′-methylenebisacrylamide (PAAm-*co*-PMBAm) copolymer via SARA ATRP is represented in Figure 1 (top). In this figure, AAm (0.8 g, 11.1 mmol), MBAm (0.19 g, 1.23 mmol), CuCl_2_ (20.8 mg, 154 μmol), Me_6_TREN (71.1 mg, 309 μmol), ECP (70.3 mg, 515 μmol), DMSO (3.13 mL), and water (3.13 mL) were added to a 10 mL Schlenk flask equipped with a magnetic stir bar. Next, a Cu^0^ wire (*l* = 10 cm; *d* = 1 mm) was added to the Schlenk flask, which was sealed with a glass stopper, deoxygenated in three freeze–vacuum–thaw cycles, and purged with nitrogen. The flask was transferred to a water bath (25 °C), and the reaction took place for 1.5 h. The final mixture was dialyzed using deionized water (c.o. = 3500), and the pure polymer was obtained after freeze-drying. The chemical structure of the polymer was confirmed by ^1^H NMR spectroscopy, and the corresponding molecular weight and dispersity were determined by SEC.

A control PAAm homopolymer was synthesized using the same procedure, but without the addition of the crosslinker (MBAm).

### 2.4. Application of the Obtained Polymers in Molecular Imprinting

The schematic representation of the assembly of the imprinted-based sensor is shown in Figure 1 (bottom) and can be essentially separated in two stages: the preparation of the working electrodes (WEs) and the imprinting stage.

The WEs were first prepared by electrochemical cleaning, under an acidic environment. This was made with a solution of H_2_SO_4_ 0.5 M that underwent 5 cycles of cyclic voltammetry (CV) procedures ranging from −0.2 to +1.2 V at 0.05 V/s. Then, the WEs were incubated in a solution of 3-MPA (10 mM) for 2 h at 25 °C, in the dark. The carboxylic acid groups present on the gold surface (Au-SPE/3-MPA) were subsequently modified with a solution of 50 mM EDAC and 25 mM NHS, for 20 min at room temperature, followed by washing with water. The protein was then bound to the surface by casting on the WE a 100 U/mL CA 15-3 solution prepared in PBS buffer, pH 7.4, overnight, at 4 °C. The Au-SPE/3-MPA/CA 15-3 film was prepared after thorough wash with water to remove the unbound CA 15-3.

The imprinting phase began by incubating overnight the Au-SPE/3-MPA/CA 15-3 electrode in 0.5 mM AMA. Then, a solution of 0.5 mM PAAm-*co*-PMBAm, 0.05 mM MBAm (crosslinker), and 0.5 mM VA-044 (initiator) was added. The polymerization was conducted at room temperature, for 3 h. The resulting polymeric film was washed with water and covered with 5 μL of a proteinase K solution (400 µg/mL, in PBS buffer, pH 7.4), overnight at room temperature. The WE was then washed and incubated with oxalic acid (0.5 M, in ultrapure water) for 1 h at room temperature.

As control, non-imprinted polymer (NIP) was assembled in the same way but without the protein. A control sensor was also built using the same construction process but replacing the well-defined copolymer by the AAm monomer in step D (Figure 1, bottom).

### 2.5. Procedures for Electrochemical Reading

A solution with a redox probe of 5.0 mM K_3_[Fe(CN)_6_] and 5.0 mM K_4_[Fe(CN)_6_] prepared in PBS buffer was used in the electrochemical assays. Electrochemical impedance spectroscopy (EIS) was used to follow up the assembly and characterize the sensors. The EIS assays were conducted at a standard potential of +0.12 V, making use of a sinusoidal potential perturbation with an amplitude of 0.01 V, over a frequency range of 0.1–100 kHz, logarithmically distributed. The impedance data were fitted to a Randle’s equivalent circuit or a similar one, using the Nova 2.1.6 software linked to the potentiostat. The elements of this circuit included the uncompensated resistance of the solution phase (R_S_), the constant phase element (CPE), the charge transfer resistance (R_CT_), and the Warburg diffusion element (W).

Before creating the calibration curve, the signal of the blank was stabilized by successive incubations of the films in a PBS buffer, each of 20 min. These measurements were repeated until the charge transfer resistance (R_CT_) was minimal, indicating that the film was stabilized and ready for the measurements. The rebinding properties of the biosensor were then evaluated. For this purpose, the sensing films were calibrated with successive additions of CA 15-3 standard solutions, in the range from 0.001 to 100.0 U/mL, prepared in PBS (pH 7.4) or in FBS diluted 1:1000 in PBS (pH 7.4), on the working electrode surface, lasting 20 min per standard solution. After each incubation, the electrodes were washed with buffer, dried, and electrochemically analyzed using an iron redox probe to make the EIS measurements. These data were crucial for determining the relationship between the measured R_CT_ and the concentration of CA 15-3. After each calibration, the electrode was discarded.

The selectivity studies were conducted for CEA (2.5 ng/mL), CA 125 (35 U/mL), glucose (0.7 mg/mL), and urea (0.2 mg/mL), using a competitive assay in which CA 15-3 was present (30 U/mL). All solutions were prepared in PBS pH 7.4, and the analysis was performed in triplicate.

## 3. Results and Discussion

### 3.1. Synthesis and Characterization of the PAAm-co-PMBAm Copolymers

**Figure 2:**
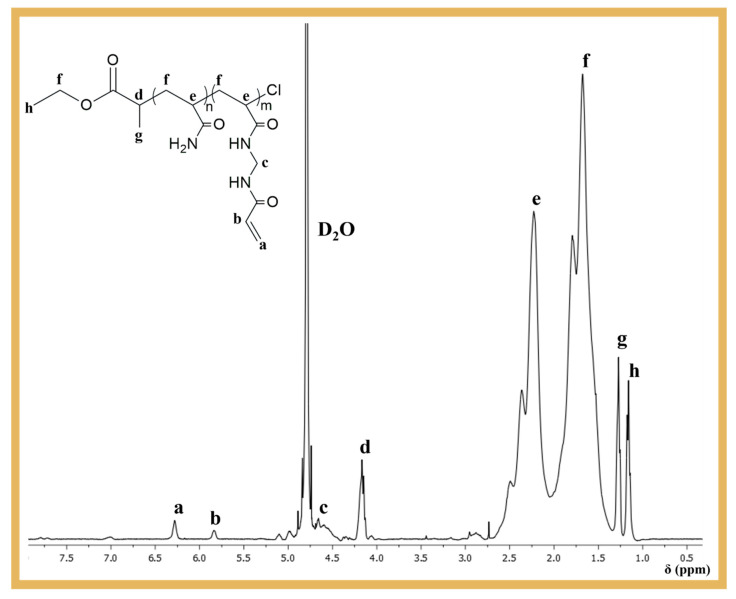
400 MHz ^1^H NMR spectrum in D_2_O of the purified P(AAm_23_-co-MBAm_3_) copolymer obtained by SARA ATRP.

The copolymerization of AAm and MBAm (Figure 1) was carried out by SARA ATRP in DMSO/water = 50/50 (*v*/*v*), using a molar ratio of [AAm]_0_/[MBAm]_0_/[ECP]_0_/[CuCl_2_]_0_/[Me_6_TREN]_0_ = 23/3/1/0.3/0.6. The aim was to introduce pendant double bonds into the PAAm structure, which could be used as crosslinking sites during the MIP process. After purification, the polymer was analyzed by ^1^H NMR spectroscopy to confirm its chemical structure and the presence of the pendant vinyl groups at ~5.75–6.5 ppm (Figure 2), which were essential for the development of the biosensor proposed in this work.

The obtained PAAm-*co*-PMBAm copolymer was also characterized by SEC to determine the number-average molecular weight (*M*_n_) and dispersity (*M*_w_/*M*_n_). The chromatograms obtained (see Figure S1) showed a bimodal molecular weight distribution with high dispersity (*M*_w_/*M*_n_ > 1.5), which could indicate poor control over the polymerization of PAAm. However, considering that the reaction contained a crosslinker, and the NMR analysis revealed a lower percentage of pendant double bonds than expected (one vinyl group for every five copolymer chains), the population of higher molecular weight polymer chains could be due to some crosslinking events. To test this hypothesis, a control PAAm homopolymer was prepared under the same polymerization conditions as the ones employed for the copolymer, but without the crosslinker. As shown in Figure S1, the molecular weight distribution of PAAm was monomodal and narrow (*M*_w_/*M*_n_ = 1.16), indicating an excellent control over the polymerization. It was also consistent with the population of lower molecular weight PAAm-*co*-PMBAm chains, confirming the hypothesis of partial crosslinking during polymerization.

It is also worth noting that ATRP provides a degree of control and is the preferred method for producing such polymers. To prove this, an analogous PAAm-*co*-PMBAm copolymer was synthesized by free-radical polymerization, under the same conditions as in ATRP (temperature and concentration), using 3% (molar) of the initiator (VA-044) relative to the PAAm monomer. The results showed that after only 120 s of polymerization, a highly cross-linked polymer (insoluble) was formed.

### 3.2. Fabrication of the Biosensor

The biosensor was constructed in five steps (as described in Figure 1, bottom). These included (A) formation of the carboxylic layer on the working electrode; (B) activation of the carboxylic groups on the surface for the covalent binding of amine groups in CA 15-3; (C) blocking of the nonspecific interaction and creation of binding sites on the protein; (D) formation of the polymeric matrix around the protein in the presence of the well-defined copolymer and cross-linking agent, and (E) extraction of the proteins from the imprinted sites. After each step, the behavior of the anionic electrochemical probe [Fe(CN)_6_]^3−/4−^ was checked by EIS to confirm the occurrence of chemical modifications.

#### 3.2.1. Immobilization of the Target Molecule

The first steps were aimed at stably immobilizing the protein on the surface of the WE (Figure 1A,B). In this process, 3-MPA was bound to the WE via -SH bonds and was expected to self-assemble into a monolayer with the carboxyl groups exposed on the surface. Then, the carboxylic groups were activated to subsequently bind to the amine groups of the protein, under quasi-physiological conditions. This process was carried out by the conventional EDAC/NHS chemical reaction. This reaction is well known and forms a highly reactive *o*-acylisourea intermediate that reacts rapidly with NHS to produce a more stable succinimidyl ester intermediate [23]. This ester performs a nucleophilic substitution for any readily available amine group (on the target molecule), resulting in the formation of an amide bond between the 3-MPA-modified electrode surface and the protein.

**Figure 3:**
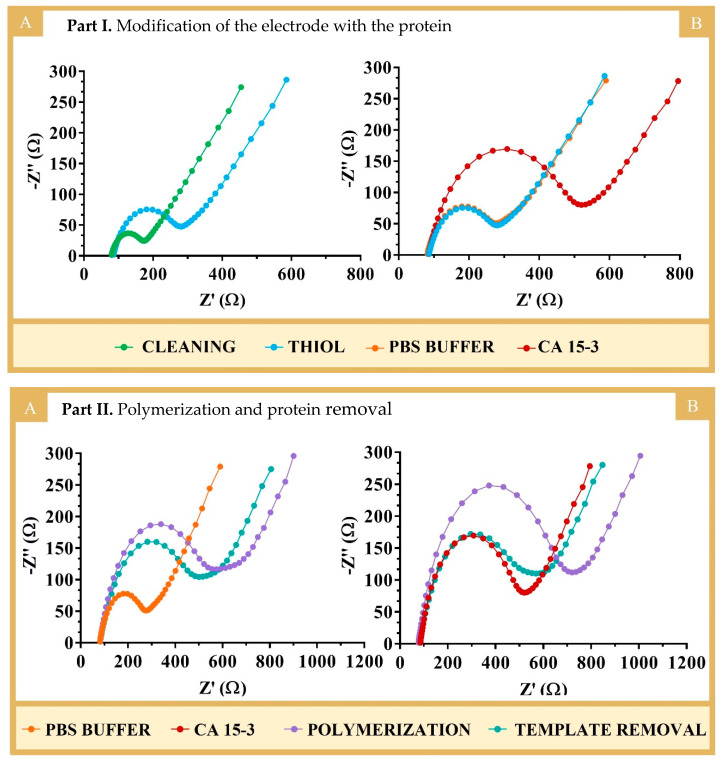
Nyquist plots obtained by EIS with 10 mM [Fe(CN)_6_)]^3−/4−^ in PBS 0.1 M, pH 7.4, at the various stages of biosensor assembly. **PART I**, until protein immobilization assembly, including (**A**) electrochemical cleaning with H_2_SO_4_ and formation of the 3-MPA layer on the working electrode (Au-SPE/3-MPA), followed by (**B**) protein binding (in the MIP) or buffer incubation (in the NIP). **PART II**, involving polymerization and template removal of the NIP (**A**) and MIP (**B**) sensors.

The successful binding of 3-MPA with the protein was confirmed by the increasing semicircles in the EIS spectra (Figure 3, PART I). In EIS, the semicircle of the Nyquist diagram for higher frequencies corresponds to the electron transfer process, while the linear part of the lower frequency corresponds to diffusion. The bare Au-SPE electrode showed a low semicircle region. The EIS spectra showed a low charge transfer resistance (R_CT_ = 94 Ω), suggesting a fast charge transfer between the redox probe and the electrode. After immobilization of 3-MPA, the value of R_CT_ increased to 197 Ω, indicating the successful modification of Au-SPE by blocking the charge transfer at the interface (Figure 3, PART I(A)). These results were generally in agreement with previous studies in the literature [14], including works involving the binding of 3-MPA to gold electrodes [24]. In terms of protein binding (Figure 3, PART I(B)), there was also an increase in the R_CT_ value (420 Ω) compared to that obtained from the incubation with PBS, which showed no change in the R_CT_ signal. The increase observed in protein binding reflected the presence of a non-conducting material (which is the case for proteins). Thus, the overall EIS data demonstrated the formation of the Au-SPE/3-MPA layer and the subsequent binding of the protein to the Au-SPE/3-MPA surface.

#### 3.2.2. Polymerization and Template Removal

The first phase of this process was to prepare the surface so that it could stably receive the imprinted polymer. This began with the incubation of the AMA monomer on the electrode, which combined amine and vinyl groups in the same structure (Figure 1C). The amine groups bound to the carboxyl groups that remained active in the previous stage, preventing side reactions in the next stage of electrode modification. Some of the amine groups also formed ionic interactions with the chemical functions on the outer surface of the protein that had a negative polarity or charge. The vinyl groups on AMA, distributed over the entire electrode surface, contributed to the covalent bonding of the polymeric network to the substrate, increasing the stability of the polymeric layer on the electrode.

Polymerization was achieved by mixing vinyl-based species at the electrode surface with an initiator (VA-44), which allowed for the formation of a thin film of polymeric network around the protein to be imprinted. In the controlled MIP assembly, this mixture contained PAAm-co-PMBAm as the monomer and MBAm as the crosslinker in addition to the initiator. To confirm that the use of a controlled polymer assembly favored the AAm performance of the MIP-based biosensor, another sensor was prepared using acrylamide as the monomer.

The polymerization occurring at the WE was characterized/confirmed by EIS using 10 mM [Fe(CN)_6_]^3−/4−^ in PBS at pH 7.4 (Figure 3, PART II). Figure 3, PART II, shows the Nyquist plots for the formation of the polymers NIP (A) and MIP (B). Overall, an increase in the semicircle was observed for both MIP and NIP after polymerization, indicating the formation of the polymer, which was non-conductive and behaved like an insulating layer. As expected, polymerization with AAm (Figure S2) led to a similar result.

The preparation of the MIP was completed after the extraction of the imprinted protein, as soon as the polymerization was finished. This was necessary to expose the imprinted sites and allow for the binding of the target molecules [24]. It is well known that target molecules trapped in the polymer matrix are often difficult to remove, especially in the case of imprinted proteins, due to their size and complex structure. Therefore, various strategies have been proposed to remove proteins from the polymer matrix, including changing the pH or ionic strength, using detergents, electrode potential, elevated temperatures, or enzymatic action [25,26]. Overall, the use of enzymatic and chemical measures can favor the process of protein removal. Proteolytic enzymes cleave peptide bonds under mild conditions and contribute to the extraction of proteins from a polymer matrix. However, peptide fragments may remain tightly bound to the polymer network. In this case, chemical methods using acids or bases can aid protein/peptide removal. The most appropriate chemical compound for this purpose must ensure the integrity of the polymer and its subsequent stability.

In this work, proteinase K (400 µg/mL) was used first, with the electrodes incubated overnight. This long exposure time should favored the complete removal of the protein. However, a small decrease in R_CT_ was observed, which indicated the incomplete removal of the target molecule, as shown in Figure S3A,B. The incomplete removal of the protein was also confirmed by calibrating the corresponding MIP and NIP electrodes; the electrodes were not very sensitive to the presence of standard solutions of the protein, as shown in Figure S3C,D.

The protein removal process was then completed by incubating the electrodes with an oxalic acid solution (0.5 M) for 1 h at room temperature. This resulted in a further decrease in the R_CT_ values (Figure 3, PART II), indicating the formation of voids in the imprinted structure, which reduced the insulating properties of the original polymer film. A slight decrease in R_CT_ was also observed at the NIP electrode (which underwent the same “protein removal” procedure as the MIP, because it acted as a control), possibly due to the partial chemical degradation and/or modification of the polymer surface or the leaching of oligomer fragments that were not firmly attached to the polymer network.

### 3.3. Physicochemical Characterization of the Biosensor

**Figure 4:**
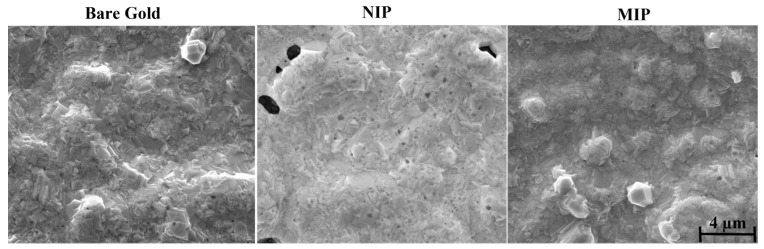
Results of the SEM analysis of bare gold, MIP, and NIP films.

Surface Electron Microscopy (SEM) is a valuable tool for the morphological analysis of materials and can generate three-dimensional, high-resolution images. To evaluate the modification of the electrode surface with MIP and NIP films on the surface of the working electrodes, the morphology before and after polymerization was examined by SEM (Figure 4).

When the gold SPEs were modified with the MIP and NIP materials, a change in the topographical aspect of the electrode was expected. At least, the MIP and the NIP showed a different appearance from that of the control, meaning that some additional layer existed on the top of each electrode. For the NIP material, the brightness in the modified SPE was more evident than for the MIP film. In principle, this could reflect a higher electron conductivity for the MIP, but this could be a local artifact, as electron microscopy is not an adequate tool for this study. As an additional confirmation of this modification, the surfaces of the gold electrodes appeared smoother and less shiny after the chemical modifications, which could be related to the deposition of a polymer film.

### 3.4. Analytical Response of the Electrochemical Biosensor

**Figure 5:**
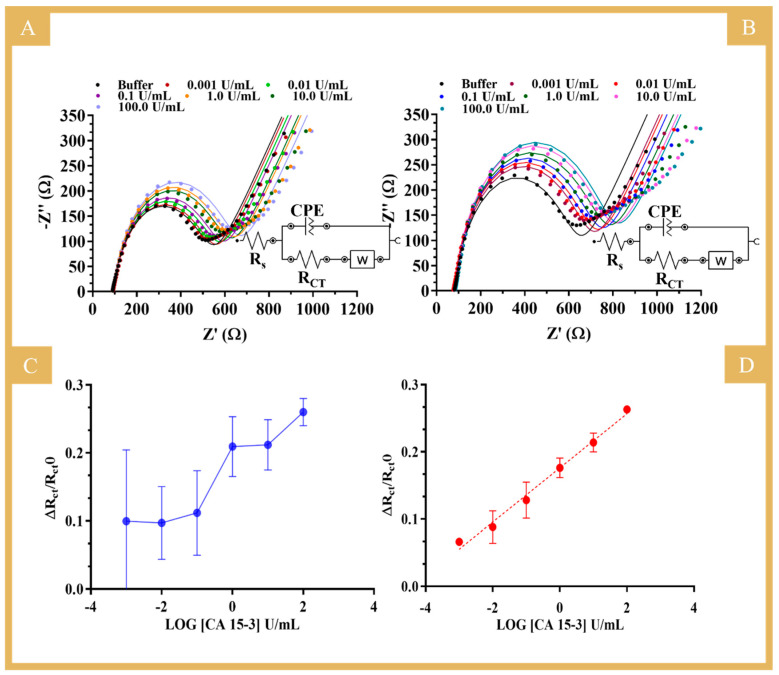
(**A**,**B**) Nyquist plots of EIS measurements of NIP*s* and MIPs, respectively, in 5 mM [Fe(CN)_6_]^3−^ and 5 mM [Fe(CN)_6_]^4−^ in PBS buffer with different concentrations of CA 15-3. (**C**,**D**) The corresponding calibration curves of NIP and MIP (Y = 0.0403X + 0.1761; R^2^ = 0.9901), respectively. The Nyquist plots evidenced a linear behavior for ΔR_CT_, equal to (R_CT/standard_ − R_CT/blank_)/R_CT/blank_, against log(concentration) from 0.001 U/mL up to 100 U/mL of CA 15-3 in PBS. Inset circuit: the uncompensated resistance of the solution phase (R_S_), the constant phase element (CPE), the charge transfer resistance (R_CT_), and the Warburg diffusion element (W).

The electroanalytical performance of the biosensor was evaluated by EIS measurements in the presence of CA 15-3 standard solutions at different concentrations (data in Table S1). As for the R_CT_ values, both NIP (Figure 5A) and MIP (Figure 5B) showed an increasing trend of resistance with the increase in protein concentration. Figure 5C,D show the calibration curves of NIP and MIP, respectively, (ΔR_CT_/R_CT_0 versus log [CA 15-3]), with a linear range from 0.001 to 100 U/mL for the MIP-based sensor. These tests were performed in a PBS buffer and yielded, respectively, the following regression equation and correlation coefficient for MIP: R_CT_ = 0.0403 (log [CA 15-3, U/mL]) + 0.1761 and 0.9901. The RSD of the MIP readings was up to 10%. The NIP did not show a linear response for CA 15-3 in the concentration range analyzed, and the data obtained were largely random (RSD up to 36%), as indicated by the observed high standard deviation. This suggests that the main binding mechanism to the MIP was related to the presence of the imprinted CA 15-3 cavities (sites that behaved like natural antibodies) within the polymer matrix.

The linearity range and lower limit of the linear range (LLLR) reported in the literature using MIP sensors for CA 15-3 determination and the results of this work are compared in Table S2. The LLLR is the lower concentration of the linear response observed, meeting an R-squared requirement for the datapoints considered (six standard solutions) of at least 0.99. Compared to the other methods, this work provided the best LLLR, which allowed for the quantification of CA 15-3 at a level as low as 0.001 U/mL, which is well below the threshold needed for the clinical assessment of breast cancer progression and recurrence (cut-off value of 30 U/mL).

### 3.5. Comparison to an “Uncontrolled” Electrochemical MIP Biosensor

To confirm the advantages of a controlled polymerization step in ATRP polymerization, another biosensor was prepared using a MIP material formed by uncontrolled radical polymerization of AAm. The results obtained for this electrode are shown in Figure S4. In general, a narrower linear response range was observed for CA 15-3, but the most important indication was the high standard deviation obtained for all standard solutions. This indicated a high variation between the different electrodes, both in background signals and in response sensitivity. The signals from R_CT_ were considered as a ratio to the blank, which in principle reduced the variation between different units, but in this case, the standard deviation values were significant and similar for both NIP and MIP. These results indicated that the incubation of CA 15-3 with PAAm-co-PMBAm prior to polymerization created a different chemical/stereochemical environment around the protein that distinguished it from the other regions of the polymer. This specific complex formation is the main innovation of this work compared to other previously published MIP materials and has proven to be very important as it favors specific binding over non-specific binding. The fact that the small copolymer was obtained through a controlled process is also crucial, as this allowed for the production of polymer structures that were highly reproducible in terms of their physical and chemical properties, thereby ensuring a better reproducibility in terms of analytical output. As such, the use of advanced polymerization techniques to produce well-defined polymers for MIP sensors offers advantages in terms of linear response range and helps to reduce LLLR 10-fold and to reduce the variability of the signals obtained with different electrodes.

Overall, the copolymer obtained with a pre-incubation of protein/PAAm-co-PMBAm showed much better results in terms of reproducibility, when compared to the MIP assembled without this stage. Thus, the controlled polymerization approach proposed here improved the detection capacity of the final MIP and, importantly, the reproducibility of the sensor units.

### 3.6. Selectivity Study

The selectivity study evaluated the ability of the biosensor to discriminate a specific target molecule in a sample containing various other molecules with similar or different structures at different and/or random concentrations. In this work, the EIS response of solutions containing a possible interfering substance and a concentration of CA 15-3 set to 30 U/mL was compared to the response of a standard solution containing only CA 15-3 at the same concentration. Each solution was prepared in 1000-fold diluted serum and incubated on the sensor surface for approximately 20 min, the time used to calibrate the biosensor with CA 15-3 standard solutions. The interfering substances selected for this purpose were normal components of serum. They were CEA, CA 125, glucose, and urea at concentrations of 2.5 ng/mL, 35.0 U/mL, 0.7 mg/mL, and 0.2 mg/mL, respectively.

**Figure 6:**
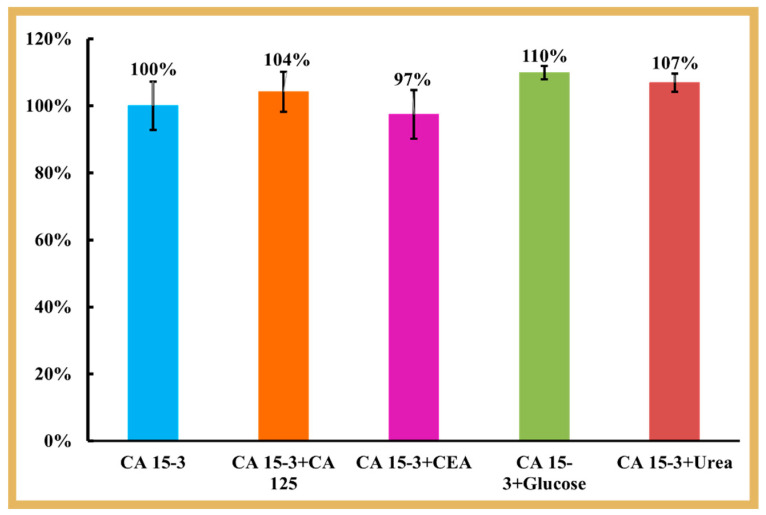
Selectivity studies using the binary solutions method. The interfering species studied were CEA, CA 125, glucose, and urea.

The selectivity test was performed by incubating in one MIP biosensor unit the standard solution of CA 15-3 and in another MIP biosensor unit the solution containing CA 15-3 (at the same concentration) and possible interfering species. The signals obtained were compared and plotted, as shown in Figure 6. The comparison of the R_CT_ values revealed that the CEA protein (4%) and the CA 125 protein (3%) binding was negligible when competing with the primary compound. For glucose and urea, the interference values were higher, reaching 10% and 7% respectively, probably due to the much higher concentration of these substances in serum.

Overall, the results obtained showed good selectivity in the detection of CA 15-3 in a binary solution, i.e., the sensor showed high affinity for CA 15-3 in the presence of other co-existing molecules.

### 3.7. Application to CA 15-3 Detection in Serum

After the behavior in PBS was investigated, the biosensor was evaluated in a more realistic environment. For this purpose, tests were carried out with standard solutions of CA 15-3 prepared in FBS and ranging from 0.001 to 100 U/mL. FBS is a complex matrix very similar to human serum, so that the performance of the biosensor can be mimicked under real sample analysis conditions.

**Figure 7:**
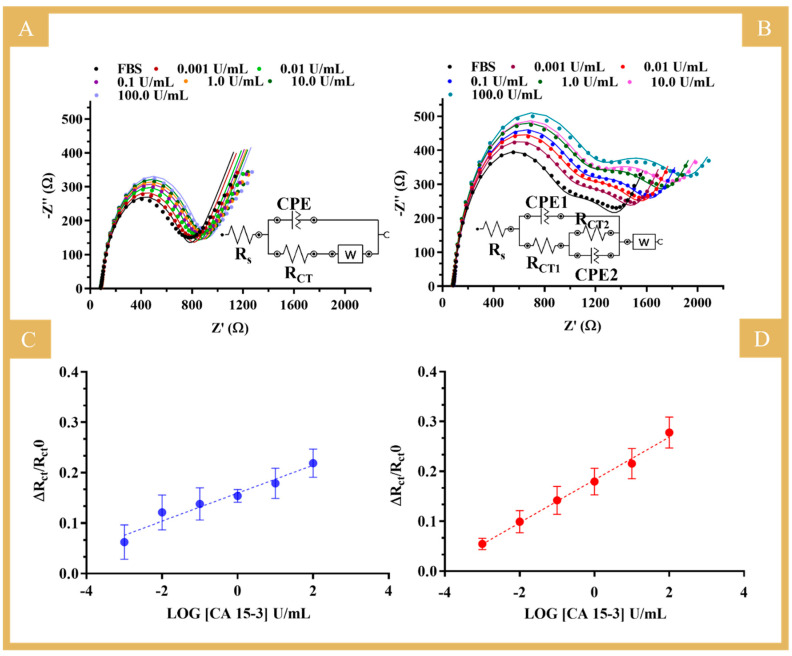
Nyquist plots of the EIS measurements of NIP- (**A**) and MIP (**B**) -based biosensors and the corresponding calibration curves (**C**,**D**), respectively, in 5.0 mM [Fe(CN)_6_]^3−^ and 5.0 mM [Fe(CN)_6_]^4−^ by increasing CA 15-3 in FBS diluted in buffer. (**C**,**D**) Calibration curves with the respective error bars for triplicates of the MIP (Y = 0.0429X + 0.1829; R^2^ = 0.9935) and NIP (Y = 0.0277X + 0.1593; R^2^ = 0.9551). Inset circuit: uncompensated resistance of the solution phase (R_S_), constant phase element (CPE), charge transfer resistance (R_CT_), the Warburg diffusion element (W).

The results obtained under these conditions are shown in Figure 7 for the NIP (A and C) and the MIP biosensors (B and D) and in Table S1. The equivalent circuit used to fit the two semicircles observed in the MIP calibration is shown in Figure 7B, inset [27]. The first semicircle is related to the binding of the protein to the imprinted sites on the polymer, as it is a similar behavior to that observed in the calibrations using buffer. Thus, for comparison purposes, the calibrations in serum used the relative data of R_CT1_ against the logarithmic concentration. The second semicircle observed was the same for all electrodes tested under the same conditions and could be related to the protein incubation in the construction stage, as it was different from previous constructions (Figure S5). So, we hypothesized that the commercial protein might partially aggregate with time (although stored properly), as the response suggested inhomogeneity, and this was consistent across the different units. This affected the subsequent stages of the design and resulted in two semicircles when the protein was removed that were further intensified upon calibration with increasing concentrations of the standard solutions.

In general, the MIP sensor showed a trend in a linear range from 0.001 to 100 U/mL, with a sensitivity similar to that in buffered solutions (average slope of 0.0429 versus 0.0403) and a good linear trend (squared correlation coefficient of 0.9935). The NIP showed a tendency of increased R_CT_ values as the concentration of CA 15-3 increased, but the quality of the linear trend was very limited (squared correlation coefficient of 0.955). The more reproducible response obtained in diluted FBS (compared to PBS alone) was probably related to the ion content of the serum samples. FBS contains many different components, from macro to small molecules, which are inherently charged and whose effect on the EIS response is certainly different from that of PBS. It is apparent that the presence of more ions of large structural diversity justifies the more stable electrical readings in both MIP and NIP sensor materials. Overall, the MIP sensor calibrated in FBS showed particularly good analytical performance, comparable in slope and linear range to that obtained in PBS. Moreover, this calibration in serum was found very important as it favored the accuracy of the analytical data obtained by the direct analysis of real samples.

In summary, given the good analytical properties of the biosensor in complex matrices, the results obtained are quite promising, which is particularly relevant for applications in the PoC context, as the sensor was able to detect the presence of the target molecule (CA 15-3) in a complex matrix at a level 1000 times lower than the detectable physiological level.

## 4. Conclusions

This work reports a novel approach for the preparation of MIP materials using SARA ATRP, showing a linear response for a wide concentration range of CA 15-3 and increased reproducibility of the different electrode units. This was confirmed by comparison with an equivalent MIP prepared by conventional radical polymerization. In particular, the novel biosensor showed excellent performance, with a wide linear response range, down to 0.001 U/mL, which was 10 times better than that achieved with the conventional polymerization technique; it also showed much better reproducibility (this linear response range was used for 1000× diluted samples). This improved performance of the MIP material is inextricably linked to the complex formation prior to polymerization and the specific use of PAAm-co-PMBAm in this complex phase, which enabled a better stereochemical recognition of CA 15-3 at the imprinted binding sites. It is important to note that this copolymer, PAAm-co-PMBAm, was produced by controlled radical polymerization processes, which could also be related to the better reproducibility of the MIP materials compared to the NIP materials.

Overall, the biosensor produced with the MIP obtained by controlled growth showed excellent analytical results for the detection of CA 15-3 in serum samples. In addition to its analytical properties, the resulting analytical approach is portable, inexpensive, and easy to implement and has good selectivity properties. The MIP setup presented here can be extended to the construction of other MIP materials for other protein-based biomarkers that signal health/disease states. This is a contribution to the further development of the current PoC analysis scenario in relation to cancer diagnosis and monitoring.

## Data Availability

Data are contained within the article.

## Supplementary Materials

The following supporting information can be downloaded at: https://www.mdpi.com/article/10.3390/bios14050222/s1, see refs. [28,29,30,31].
