# Supplementary material for: An Innovative Approach for Tailoring Molecularly Imprinted Polymers for Biosensors—Application to Cancer Antigen 15-3
Source: Biosensors (Basel). 2024 Apr 30;14(5):222. doi: 10.3390/bios14050222 (PMC11117626; doi:10.3390/bios14050222)
Supplement: Supplementary file 1 [file biosensors-14-00222-s001.zip › biosensors-2914095-supplementary.pdf]

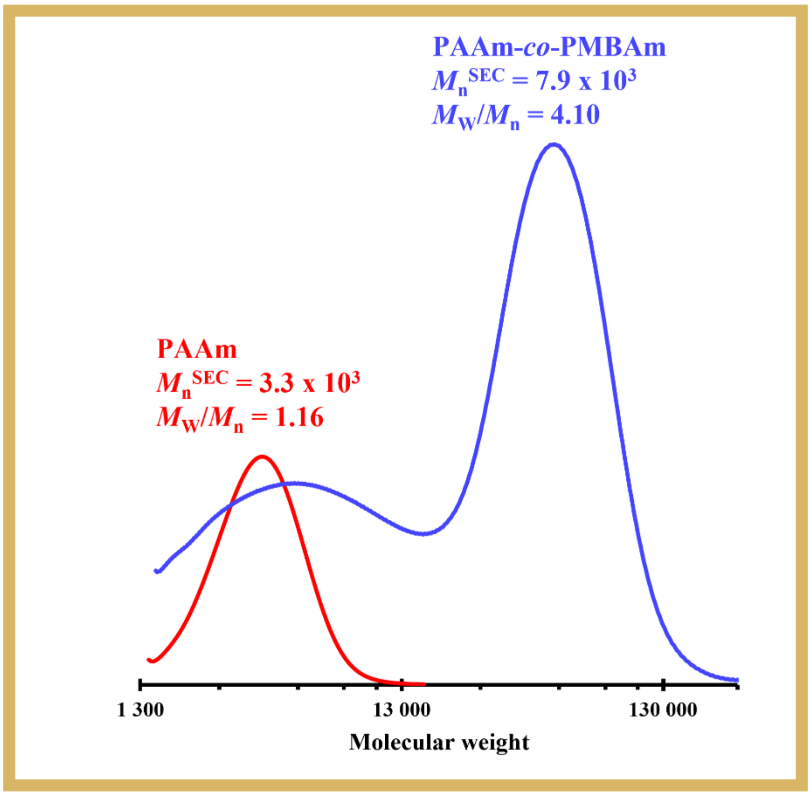

Figure S1 – Molecular weight distribution (normalized RI signal) of PAAm homopolymer and PAAm-*co*-PMBAm copolymer, obtained by SEC using 0.1 M Na_2SO_4 (aq)/1 wt% acetic acid/0.02% NaN_3 as the eluent.

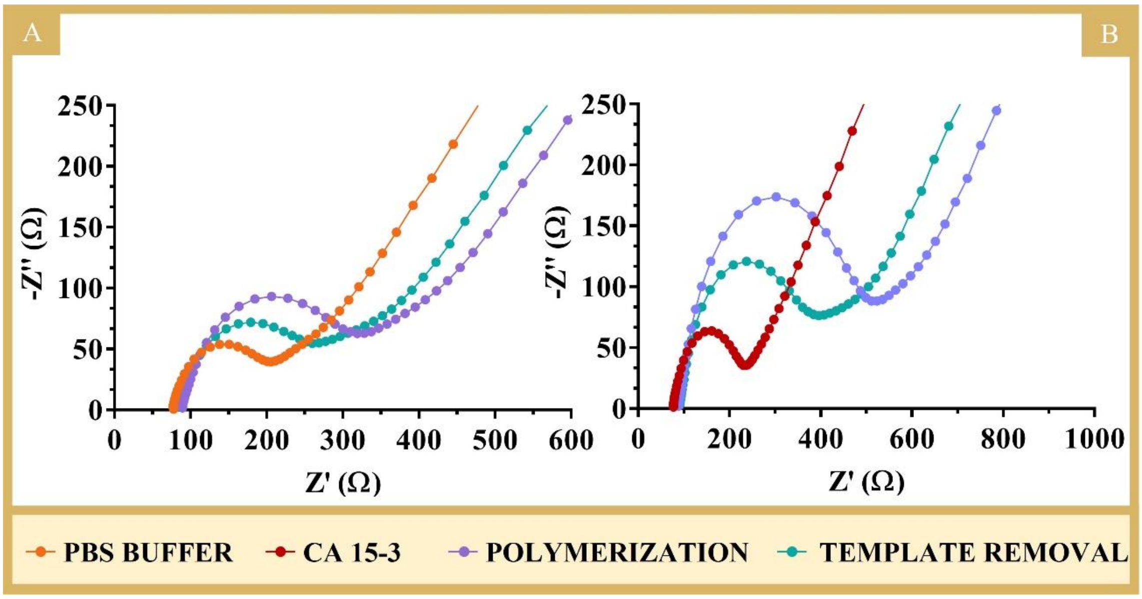

Figure S2 – Characterization of polymerization and template removal of the NIP (A) and MIP (B) sensor. EIS Nyquist diagrams in 10 mM $[Fe(CN)_6)]^{3-/4-}$ in PBS buffer 0.1 M, pH 7.4.

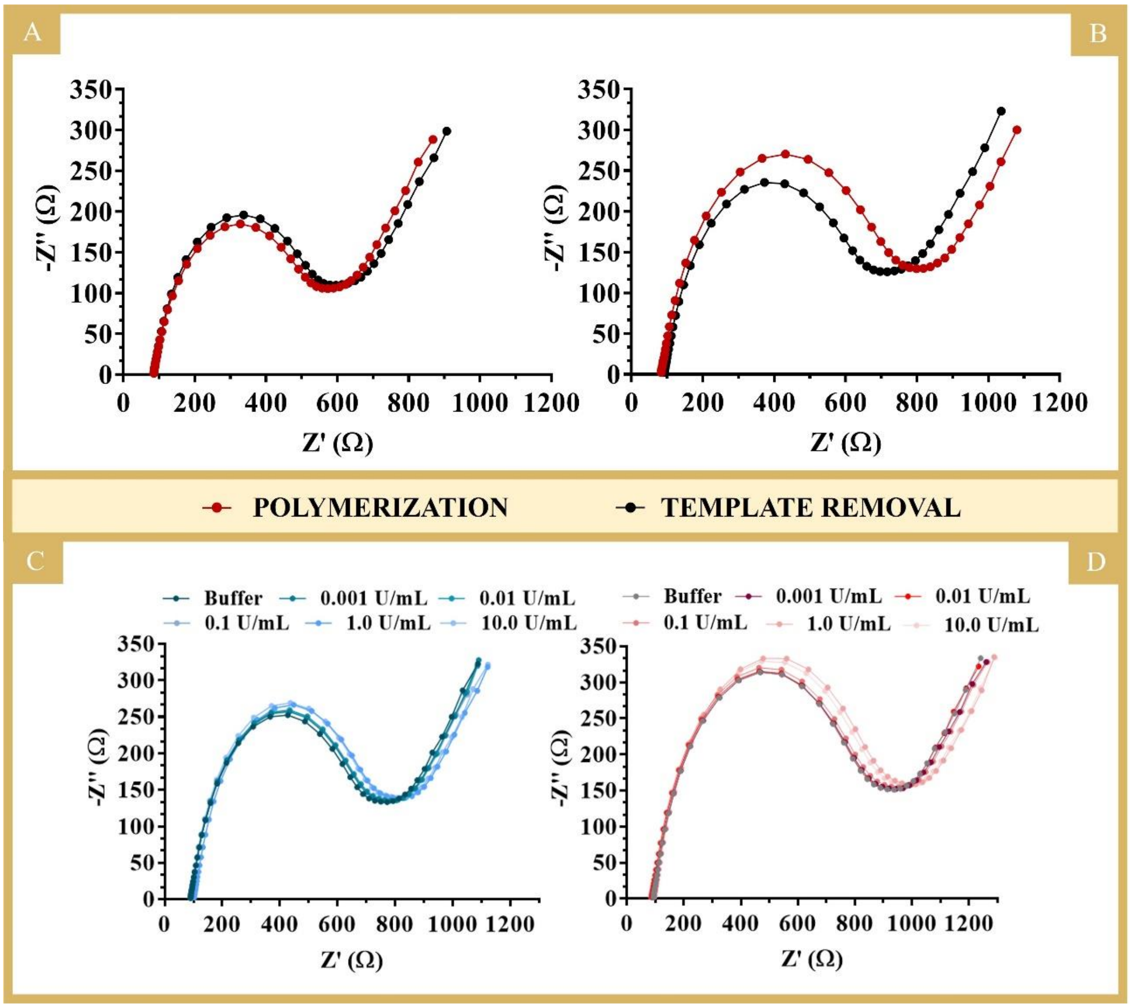

Figure S3 – Characterization of polymerization and template removal of the NIP (A) and MIP (B) sensor. Analytical response of the NIP (C) and MIP (D) sensors. EIS Nyquist diagrams in 10 mM $[Fe(CN)_6)]^{3-/4-}$ in PBS buffer 0.1 M, pH 7.4.

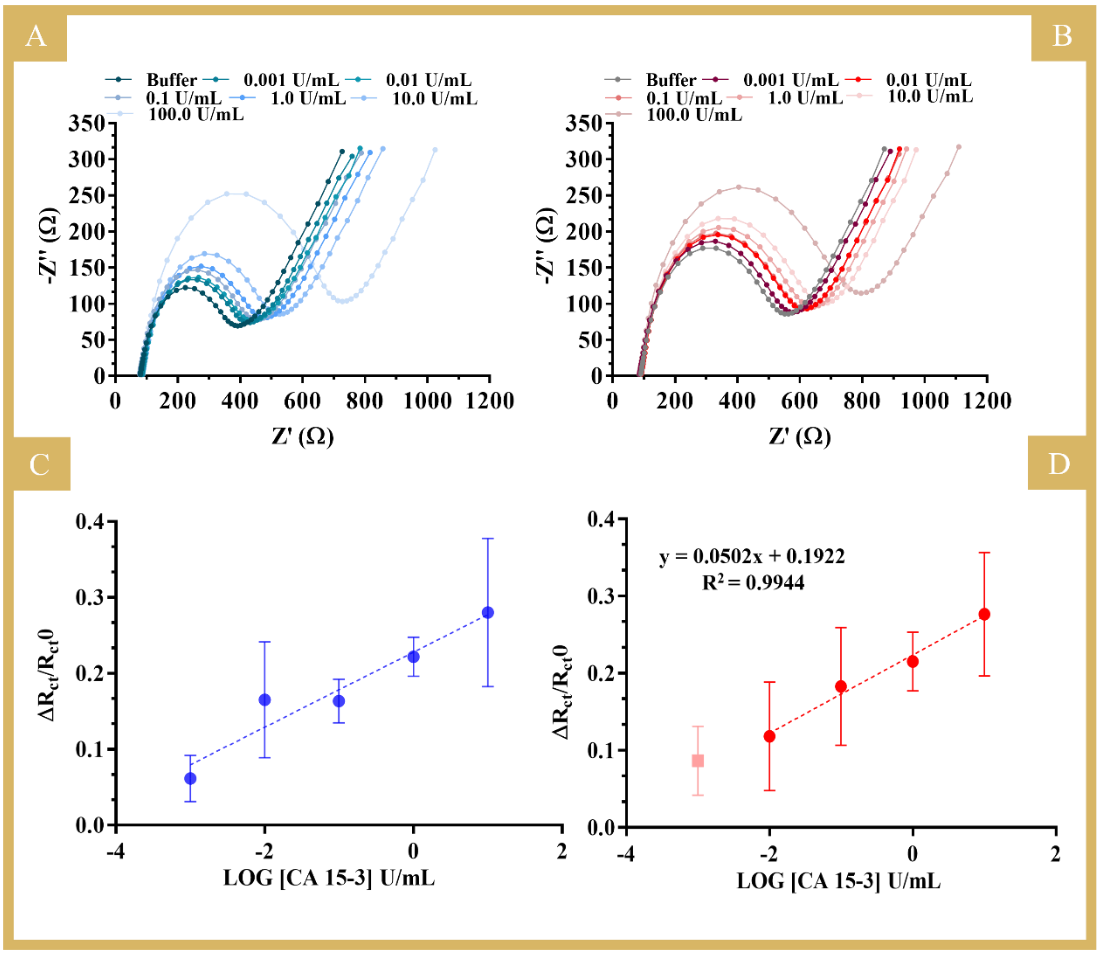

Figure S4 – (A) and (B) EIS measurement of NIPS and MIPs, respectively, in 5 mM $K_3[Fe(CN)_6]^{3-}$ and 5 mM $K_4[Fe(CN)_6]^{4-}$ in PBS buffer with different concentrations of CA 15-3. (C) and (D) The corresponding calibration curves of NIP and MIP, respectively.

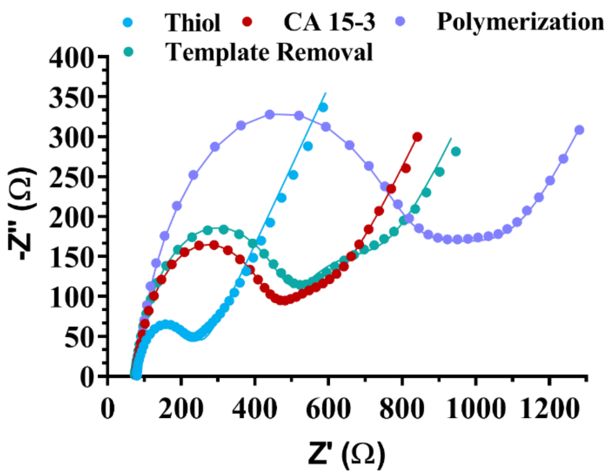

Figure S5 – Nyquist plots of the different stages of the MIP construction. Data corresponding to the specific electrode used in the calibration in serum (Figure 7 of the main manuscript). EIS Nyquist diagrams in 10 mM $[Fe(CN)_6)]^{3-/4-}$ in PBS buffer 0.1 M, pH 7.4.

Table S1. List of standard solutions and the corresponding mean and standard deviations, in the corresponding studies (as calculated).

Calibrations in buffer		
NIP	$\Delta R_{CT}/R_{CT}0$ (n=3)	SD (n=3) / Error bar
0.001 U/mL	0.0996507	0.085736
0.01 U/mL	0.0970483	0.043546
0.1 U/mL	0.1119657	0.050676
1.0 U/mL	0.2092808	0.036075
10.0 U/mL	0.2118485	0.030271
100.0 U/mL	0.2601951	0.016388
MIP	$\Delta R_{CT}/R_{CT}0$ (n=3)	SD (n=3) / Error bar
0.001 U/mL	0.0663022	0.004411
0.01 U/mL	0.0880527	0.019621
0.1 U/mL	0.1281479	0.021844
1.0 U/mL	0.1760057	0.012011
10.0 U/mL	0.2139300	0.011377
100.0 U/mL	0.2631117	0.003427
**Calibrations in diluted serum**		
NIP	$\Delta R_{CT}/R_{CT}0$ (n=3)	SD (n=3) / Error bar
0.001 U/mL	0.062544	0.027752
0.01 U/mL	0.121017	0.028090
0.1 U/mL	0.137756	0.026098
1.0 U/mL	0.153735	0.010524
10.0 U/mL	0.178802	0.024636
100.0 U/mL	0.218767	0.022992
MIP	$\Delta R_{CT}/R_{CT}0$ (n=3)	SD (n=3) / Error bar
0.001 U/mL	0.054862	0.009289
0.01 U/mL	0.099178	0.017912
0.1 U/mL	0.141831	0.022774
1.0 U/mL	0.179492	0.021719
10.0 U/mL	0.215402	0.024956
100.0 U/mL	0.277863	0.025463

Selectivity assays	
Solution components	SD (n=3) / Error bar
CA 15-3	0.072345
CA 15-3+CA 125	0.059541
CA 15-3+CEA	0.072727
CA 15-3+Glucose	0.019555
CA 15-3+Urea	0.026814

Table S2 – Analytical performance of sensor reported in the literature for the detection of CA 15-3.

Sensing Approach	Transducer	Response Range U/mL	Limit of detection U/mL	Reference
Sensor MIP	Electrochemical	0.25-10	0.05	[1]
Sensor MIP	Electrochemical	0.10-100	0.10	[2]
Sensor MIP	Electrochemical	5-50	1.50	[3]
Sensor MIP	Electrochemical	5-35	1.16	[4]
Sensor MIP	Electrochemical	0.001-100	---	This Work

References

1. Gomes, R.S., F.T.C. Moreira, R. Fernandes and M.G.F. Sales, Sensing CA 15-3 in point-of-care by electropolymerizing O-phenylenediamine (oPDA) on Au-screen printed electrodes, PLOS ONE. 13 (2018) e0196656. https://doi.org/10.1371/journal.pone.0196656.
2. Ribeiro, J.A., C.M. Pereira, A.F. Silva and M.G.F. Sales, Disposable electrochemical detection of breast cancer tumour marker CA 15-3 using poly(Toluidine Blue) as imprinted polymer receptor, Biosensors and Bioelectronics. 109 (2018) 246-254. https://www.sciencedirect.com/science/article/pii/S095656631830174X.
3. Pacheco, J.G., M.S.V. Silva, M. Freitas, H.P.A. Nouws and C. Delerue-Matos, Molecularly imprinted electrochemical sensor for the point-of-care detection of a breast cancer biomarker (CA 15-3), Sensors and Actuators B: Chemical. 256 (2018) 905-912. https://www.sciencedirect.com/science/article/pii/S092540051731907X.
4. Oliveira, A.E., Pereira, A.C., Ferreira, L.F., Disposable electropolymerized molecularly imprinted electrochemical sensor for determination of breast cancer biomarker CA 15-3 in human serum samples, Talanta, 252 (2023) 123819. https://doi.org/10.1016/j.talanta.2022.123819
